# Clinical Utility of Random Anti–Tumor Necrosis Factor Drug–Level Testing and Measurement of Antidrug Antibodies on the Long‐Term Treatment Response in Rheumatoid Arthritis

**DOI:** 10.1002/art.39169

**Published:** 2015-07-28

**Authors:** Meghna Jani, Hector Chinoy, Richard B. Warren, Christopher E. M. Griffiths, Darren Plant, Bo Fu, Ann W. Morgan, Anthony G. Wilson, John D. Isaacs, KimmeL. Hyrich, Anne Barton, on behalf of the Biologics in Rheumatoid Arthritis Genetics and Genomics Study Syndicate Collaborators, P. J. Prouse, R. K. Moitra, D. J. Shawe, M. Nisar, K. Fairburn, J. Nixon, T. Barnes, M. Hui, D. Coady, D. Wright, C. Morley, G. Raftery, C. Bracewell, M. Bridges, D. Armstrong, A. J. Chuck, S. Hailwood, N. Kumar, D. Ashok, R. Reece, S. C. O'Reilly, T. Ding, L. J. Badcock, C. M. Deighton, N. Raj, M. R. Regan, G. D. Summers, R. A. Williams, J. R. Lambert, R. Stevens, C. Wilkinson, C. A. Kelly, J. Hamilton, C. R. Heycock, V. Saravanan, A. Cope, T. Garrood, N. Ng, B. Kirkham, M. Green, A. Gough, C. Lawson, D. Das, E. Borbas, T. Wazir, P. Emery, S. Bingham, H. A. Bird, P.G. Conaghan, C. T. Pease, R. J. Wakefield, M. Buch, I. Bruce, R. Gorodkin, P. Ho, B. Parker, W. Smith, E. Jenkins, C. Mukhtyar, K. Gaffney, A. J. Macgregor, T. Marshall, P. Merry, C. DeSilva, F. N. Birrell, P. R. Crook, B. Szebenyi, D. Bates, D. James, T. Gillott, A. Alvi, C. Grey, J. Browning, J. F. McHale, I.C. Gaywood, A. C. Jones, P. Lanyon, I. Pande, M. Doherty, A. Gupta, P. A. Courtney, A. Srikanth, A. Abhishek, L. Das, M. Pattrick, H. N. Snowden, A. P. Bowden, E. E. Smith, P. Klimiuk, D. J. Speden, S. Naz, J. M. Ledingham, R. G. Hull, F. McCrae, A. Cooper, S. A. Young‐Min, E. Wong, R. Shaban, A. D. Woolf, M. Davis, D. Hutchinson, A. Endean, D. Mewar, E. J. Tunn, K. Nelson, T. D. Kennedy, C. Dubois, J. Pauling, E. Korendowych, T. Jenkinson, R. Sengupta, A. Bhalla, N. McHugh, T. O'Neil, A. L. Herrick, A. K. Jones, R. G. Cooper, W. G. Dixon, B. Harrison, C. D. Buckley, D. C. Carruthers, R. Elamanchi, P. C. Gordon, K. A. Grindulis, F. Khattak, K. Raza, K. Situnayake, M. Akil, S. Till, L. Dunkley, R. Tattersall, R. Kilding, T. Tait, J. Maxwell, S. Till, K.-P. Kuet, M. J. Plant, F. Clarke, J. N. Fordham, S. Tuck, S. K. Pathare, A. Paul, C. P. Marguerie, S. P. Rigby, N. Dunn, I. Abbas, C. Filer, V. E. Abernethy, A. R. Clewes, J. K. Dawson, G. Kitas, N. Erb, R. Klocke, A. J. Whallett, K. Douglas, A. Pace, R. Sandhu, H. John, L. Shand, S. Lane, H. Foster, B. Griffiths, I. Griffiths, L. Kay, W.-F. Ng, P. N. Platt, D. J. Walker, P. Peterson, A. Lorenzi, M. Friswell, B. Thompson, M. Lee, A. Pratt, N. D. Hopkinson, C. A. Dunne, B. Quilty, J. Marks, S. Mukherjee, D. Mulherin, S. V. Chalam, T. Price, T. Sheeran, S. Venkatachalam, S. Baskar, W. Al- Allaf, F. McKenna, P. Shah, A. Filer, S. J. Bowman, P. Jobanputra, E. C. Rankin, M. Allen, K. Chaudhuri, S. Dubey, A. Price‐Forbes, J. Ravindran, A. Samanta, P. Sheldon, W. Hassan, J. Francis, A. Kinder, R. Neame, A. Moorthy, M. Bukhari, L. Ottewell, E. Palkonyai, S. Hider, A. Hassell, A. Menon, C. Dowson, S. Kamath, J. Packham, S. Dutta, S. Price, E. Roddy, Z. Paskins, D. T. O'Reilly, V. Rajagopal, S. Bhagat, C. B. Chattopadhyay, M. Green, D. Quinn, A. Isdale, A. Brown, B. Saleem, B. Foo, Z. Al Saffar, G. Koduri

**Affiliations:** ^1^ Meghna Jani, MBChB, MSc, MRCP: Centre for Musculoskeletal ResearchInstitute of Inflammation and Repair, University of Manchester, Manchester Academic Health Science CentreManchesterUK; ^2^ Hector Chinoy, MBBS, PhD, MRCP, MSc, BMedSci: Centre for Musculoskeletal ResearchInstitute of Inflammation and Repair, University of Manchester, and NIHR Manchester Musculoskeletal Biomedical Research Unit, Manchester Academic Health Science CentreManchesterUK; ^3^ Richard B. Warren, BSc, MBChB, PhD, MRCP, Christopher E. M. Griffiths, BSc, MBBS, MD, FRCP, FRCPath, FMedSci: The Dermatology CentreSalford Royal NHS Foundation Trust, University of Manchester, Manchester Academic Health Science CentreManchesterUK; ^4^ Darren Plant, PhD, Anne Barton, MBChB, PhD, FRCP: Arthritis Research UK Centre for Genetics and Genomics, Centre for Musculoskeletal ResearchInstitute of Inflammation and Repair, University of Manchester, and NIHR Manchester Musculoskeletal Biomedical Research Unit, Manchester Academic Health Science CentreManchesterUK; ^5^ Bo Fu, PhD: Centre for BiostatisticsInstitute of Population Health, University of Manchester, Manchester Academic Health Science CentreManchesterUK; ^6^ University of LeedsLeedsUK; ^7^ University of SheffieldSheffieldUK; ^8^ Institute of Cellular Medicine, Newcastle University, and NIHR Newcastle Biomedical Research CentreNewcastle‐upon‐TyneUK; ^9^ Kimme L. Hyrich, MD, PhD, FRCPC: Arthritis Research UK Centre for Epidemiology, Centre for Musculoskeletal ResearchUniversity of ManchesterManchesterUK

## Abstract

**Objective:**

To investigate whether antidrug antibodies and/or drug non‐trough levels predict the long‐term treatment response in a large cohort of patients with rheumatoid arthritis (RA) treated with adalimumab or etanercept and to identify factors influencing antidrug antibody and drug levels to optimize future treatment decisions.

**Methods:**

A total of 331 patients from an observational prospective cohort were selected (160 patients treated with adalimumab and 171 treated with etanercept). Antidrug antibody levels were measured by radioimmunoassay, and drug levels were measured by enzyme‐linked immunosorbent assay in 835 serial serum samples obtained 3, 6, and 12 months after initiation of therapy. The association between antidrug antibodies and drug non‐trough levels and the treatment response (change in the Disease Activity Score in 28 joints) was evaluated.

**Results:**

Among patients who completed 12 months of followup, antidrug antibodies were detected in 24.8% of those receiving adalimumab (31 of 125) and in none of those receiving etanercept. At 3 months, antidrug antibody formation and low adalimumab levels were significant predictors of no response according to the European League Against Rheumatism (EULAR) criteria at 12 months (area under the receiver operating characteristic curve 0.71 [95% confidence interval (95% CI) 0.57, 0.85]). Antidrug antibody–positive patients received lower median dosages of methotrexate compared with antidrug antibody–negative patients (15 mg/week versus 20 mg/week; *P* = 0.01) and had a longer disease duration (14.0 versus 7.7 years; *P* = 0.03). The adalimumab level was the best predictor of change in the DAS28 at 12 months, after adjustment for confounders (regression coefficient 0.060 [95% CI 0.015, 0.10], *P* = 0.009). Etanercept levels were associated with the EULAR response at 12 months (regression coefficient 0.088 [95% CI 0.019, 0.16], *P* = 0.012); however, this difference was not significant after adjustment. A body mass index of ≥30 kg/m^2^ and poor adherence were associated with lower drug levels.

**Conclusion:**

Pharmacologic testing in anti–tumor necrosis factor–treated patients is clinically useful even in the absence of trough levels. At 3 months, antidrug antibodies and low adalimumab levels are significant predictors of no response according to the EULAR criteria at 12 months.

The introduction of anti–tumor necrosis factor (anti‐TNF) therapy transformed the treatment of rheumatoid arthritis (RA). However, up to 40% of patients with RA fail to respond to anti‐TNF treatment, because of either primary inefficacy or loss of response 1, 2, 3. When patients fail to have a response to their first anti‐TNF drug, therapeutic options may include switching to a biologic agent with a different mechanism of action, switching to an alternative anti‐TNF drug, or increasing the dose/reducing the length of time between infusions. The choice of a second‐line agent is often based on cost and local policies as opposed to an understanding of the mechanistic etiology of treatment failure. At present, no biomarkers are readily available to predict which treatments will work better for which patients, because until recently, the mechanisms underlying these responses have not received much attention 4, 5. The ability to predict nonresponse at an early stage of treatment with a biologic agent could potentially have major implications for health care economics and help to optimize patient care.

One explanation of the poor efficacy of anti‐TNF therapies is immunogenicity leading to the development of antidrug antibodies and low drug levels. Previous studies demonstrated that the presence of antibodies against anti‐TNF monoclonal antibodies reduces the response to treatment and increases the risk of treatment discontinuation 6, 7. Meanwhile, the utility of pharmacologic monitoring in clinical practice continues to be debated 8, 9. Indeed, the 2013 European League Against Rheumatism (EULAR) Task Force recommendations for the management of RA 9 included the following questions in their research agenda: “Is measurement of serum drug and/or drug antibody levels useful in clinical practice?” and “How can immunogenicity of [biologic disease‐modifying antirheumatic drugs] DMARDs explain the similarity of clinical trial data observed with both immunogenic and non‐immunogenic compounds?”

A challenge when interpreting the results of immunogenicity studies is wide variation in the reported antidrug antibody frequency, which may be related to several intrinsic patient factors and drug‐related and treatment‐associated factors, including concomitant treatment with DMARDs 10, 11. The diversity of detection methods, timing of the sample collection, as well as the presence of free drug may mask the detection of antidrug antibodies due to drug interference 6, 8. The latter concern may be addressed by performing radioimmunoassay (RIA), which is less susceptible to drug interference compared with enzyme‐linked immunosorbent assays (ELISAs) 12 and has been used successfully in a clinical setting 5, 13, 14.

To circumvent the issue of drug interference, previous studies used trough‐level serum samples to measure drug concentrations and antidrug antibody levels, obtained immediately prior to administration of the patient's next scheduled dose. For treatment with agents such as adalimumab and etanercept, which are administered subcutaneously by the patient at home, ascertainment of trough levels would most likely require a separate hospital visit after inefficacy of the drug has been determined by the clinician. The practical implications for the patient and the impact on service delivery of obtaining serum antidrug antibody trough levels and drug levels pose additional challenges in clinical practice.

The aims of this study were, first, to investigate whether the presence of antidrug antibodies and/or drug non‐trough levels predict treatment response in a large cohort of RA patients treated with adalimumab or etanercept and, second, to identify pretreatment factors that may predict antidrug antibody formation and/or drug levels that may help optimize future treatment decisions.

## PATIENTS AND METHODS

### Patients

Patients were recruited to participate in a prospective observational cohort study, the Biologics in Rheumatoid Arthritis Genetics and Genomics Study Syndicate (BRAGGSS) 15, from 60 centers across the UK between November 2008 and March 2013 (see Appendix A for the BRAGGSS collaborators). From the total cohort, 311 patients were selected based on the following inclusion criteria: a diagnosis of RA according to the American College of Rheumatology 1987 revised criteria for the classification of RA 16; active disease as indicated by a Disease Activity Score in 28 joints 17 using the C‐reactive protein level (DAS28‐CRP) of ≥5.1 despite previous treatment with at least 2 DMARDs, including methotrexate; white (Caucasian ancestry); and planned initiation of treatment with either adalimumab or etanercept. Adalimumab and etanercept were the anti‐TNF agents most commonly prescribed for the treatment of RA in the national UK cohort at the time that this study was designed. All patients were prescribed adalimumab 40 mg subcutaneously every other week and etanercept 50 mg subcutaneously every week throughout the duration of the study.

At baseline and following initiation of therapy, serum samples were collected from the patients, and disease activity was measured at 3, 6, and 12 months. Clinician and patient questionnaires (including self‐reported adherence) were completed at each time point. For the purposes of the current study, adherence was classified as previously defined 18. Therapeutic response was evaluated at months 3, 6, and 12 of anti‐TNF therapy, using the EULAR response criteria 19 and/or change in the DAS28. The latter was defined as the difference between the DAS28 after initiation of treatment and the pretreatment DAS (i.e., baseline DAS time point 3/6/12 month DAS28). Therefore, an improvement with treatment would equate to a positive change in the DAS28 and vice versa. All serum samples collected were sent to the Centre for Musculoskeletal Research for central processing, storage, and analysis. Contributing patients provided written informed consent, and the study was approved by a multicenter ethics committee (COREC 04/Q1403/37).

### Measurement of antidrug antibodies against adalimumab or etanercept and drug levels

Serum drug levels were tested in all serial samples after initiation of treatment and were measured in‐house using a sandwich ELISA according to the instructions of the manufacturer (Progenika Biopharma). The presence of antidrug antibodies against adalimumab and etanercept was determined by RIA (performed by personnel at Sanquin Diagnostic Services, Amsterdam, The Netherlands). The assay measures specific high‐avidity IgG antibodies against the drug, using an antigen‐binding test, as previously described 5, 20. Compared with ELISA, RIA is less prone to drug/rheumatoid factor (RF) interference 12, and can detect IgG4 antibodies, which have greater neutralization potential 21, 22. All serum samples obtained from patients receiving adalimumab were tested and defined as positive for anti‐adalimumab antibodies if titers were >12 arbitrary units (AU)/ml; this cutoff was determined based on previous reference serum comparisons 5. Previous studies did not detect antibodies to etanercept using RIA 13, 23, 24; however, 60 serum samples, which included samples obtained at all time points after initiation of etanercept treatment, were tested for completeness. Similarly, patients were defined as being anti‐etanercept antibody positive if the antibody level was >12 AU/ml. To establish a concentration–effect curve, all 331 patients were categorized according to drug concentrations (from low to high), correlating changes in the DAS28 across all time points.

### Statistical analysis

Between‐group comparisons were assessed by the *t*‐test for independent samples, the Wilcoxon‐Mann‐Whitney U test, or the chi‐square test, as appropriate. Nonparametric Spearman's correlations between the presence of antidrug antibodies and drug levels were determined. The generalized estimating equation (GEE) model with an identity link for longitudinal continuous outcomes was used to test the association between treatment response and drug and antibody levels, as well as the univariate association between drug levels and antidrug antibody status over 12 months. Furthermore, ordinal logistic regression was used to test the association between the EULAR response at 12 months and drug levels. Variables that were considered to be potential confounders were included in the GEE models in order to obtain adjusted estimates. To quantify the value of testing drug non‐trough levels and antidrug antibodies at 3 months as a predictor of the treatment response at 12 months, the area under the receiver operating characteristic (ROC) curve (AUC) was used. The GEE model with a logit link for binary outcomes was used to assess the predictors of longitudinal low‐drug‐level status over time. Statistical analyses were performed using Stata for Windows version 13.0.

## RESULTS

### Patient characteristics

The characteristics of the 311 patients at baseline are shown in Table 1. The majority of patients were female; 73.0% were RF positive, and 76.4% were anti***–***cyclic citrullinated peptide antibody positive. All patients had active disease at baseline (mean ± SD DAS28 5.8 ± 0.9), and the median disease duration prior to beginning treatment with a biologic agent was 8.1 years (interquartile range [IQR] 3.6–16.0). At baseline, more patients in the etanercept group were receiving hydroxychloroquine (9.3% versus 2.9% of patients in the adalimumab group; *P* = 0.02), but no other significant differences between the 2 treatment groups were observed.

**Table 1:** Demographic and clinical characteristics of the patients at baseline^a^

Characteristic	Total patient population (n = 331)	Adalimumab group (n = 160)	Etanercept group (n = 171)
Age, mean ± SD years	56.8 ± 11	56.2 ± 12	57.3 ± 11
Female sex	249 (75.5)	113 (70.6)	136 (79.5)
BMI, median (IQR) kg/m^2^	27.5 (23.8–32.8)	27.5 (23.7–32.3)	27.5 (23.9–32.2)
Disease duration, median (IQR) years	8.1 (3.6–16.0)	8.6 (3.7–17.2)	7.8 (3.5–15.5)
RF positive^b^	193 (73.0)	88 (70.4)	105/139 (75.5)
Anti‐CCP antibody positive^b^	241 (76.4)	115 (75.5)	126 (77.3)
ESR, median (IQR) mm/hour	26.0 (14.0–42.0)	28.0 (16.0–43.0)	23.5 (12.0–40.0)
CRP, median (IQR) mg/liter	11.7 (3.9–28.1)	10.5 (3.7–26.0)	12.70 (4.6–29.2)
DAS28, mean ± SD	5.8 ± 0.9	5.7 ± 0.9	5.9 ± 0.9
Erosive disease^b^	158 (65.0)	74 (64.4)	84 (65.6)
DMARD therapy			
Prior biologic agent	27 (8.2)	14 (8.8)	13 (7.6)
Methotrexate	168 (50.8)	89 (55.6)	79 (46.2)
Median (IQR) mg/week	20 (15–25)	20 (15–25)	20 (15–25)
Sulfasalazine	57 (17.2)	30 (18.8)	27 (15.8)
Median (IQR) mg/day	1,000 (1,000–2,000)	1,000 (1,000–2,000)	1,000 (1,000–2,500)
Leflunomide	26 (7.9)	10 (6.3)	16 (9.4)
Hydroxychloroquine	21 (13.1)	5 (2.9)	16 (9.3)
Intramuscular gold	6 (1.9)	1 (0.6)	2 (1.1)

a The disease‐modifying antirheumatic drugs (DMARDs) shown are those that were used most frequently by patients in this cohort. Except where indicated otherwise, values are the number (%). BMI = body mass index; IQR = interquartile range; anti‐CCP = anti–cyclic citrullinated peptide; ESR = erythrocyte sedimentation rate; CRP = C‐reactive protein; DAS28 = Disease Activity Score in 28 joints.

b Values are the number (%) of patients with nonmissing data.

### Antidrug antibodies against adalimumab, levels of adalimumab, and association with treatment response

Adalimumab levels and antidrug antibodies were measured in 414 available samples obtained from 160 patients. Antidrug antibodies against adalimumab were detected in 24.8% of patients who completed 12 months of followup (31 of 125 patients at ≥1 time point) and in 19.3% of all patients in the adalimumab group who were tested (31 of 160). The presence of antidrug antibodies was significantly associated with lower adalimumab levels (r_s_ = −0.51, *P* < 0.0001; if antidrug antibody titers were >100 AU, r_s_ = −0.66, *P* = 0.0041). Compared with antidrug antibody***–***negative patients, those who were antidrug antibody positive had lower mean adalimumab concentrations at 3, 6, and 12 months (see Supplementary Table 1, available on the *Arthritis & Rheumatology* web site at http://onlinelibrary.wiley.com/doi/10.1002/art.39169/abstract). The majority of patients with antidrug antibodies (28 of 31 [90.3%]) developed immunogenicity by 6 months, and 3 other patients developed antidrug antibodies between 6 months and 12 months. Titers of antidrug antibodies continued to increase, for up to 12 months in some patients (median antidrug antibody concentration 37 AU/ml [IQR 23***–***95] at 3 months; 48.5 AU/ml [IQR 18***–***200] at 6 months; 25 AU/ml [IQR 21***–***2,800] at 12 months). Despite the measurement of non‐trough levels, the maximum antidrug antibody concentration detected was 111,000 AU/ml at 12 months.

There were differences at baseline between patients in whom antidrug antibodies developed and those in whom antidrug antibodies did not develop, particularly with regard to the median methotrexate dosage, which was significantly higher in the latter group (Table 2). The median disease duration prior to commencing treatment with a biologic agent was almost twice as long in patients with antidrug antibodies compared with those without antidrug antibodies (14.0 years [IQR 6.7–19.4] versus 7.7 years [IQR 3.6–16.04]; *P* = 0.03).

**Table 2:** Baseline characteristics of the patients receiving adalimumab, according to the presence or absence of antidrug antibodies^a^

Characteristic	Anti‐adalimumab antibodies (n = 31)	No anti‐adalimumab antibodies (n = 129)
Age, mean ± SD years	56.0 ± 11	56.2 ± 12
Female sex	21 (67.7)	92 (71.3)
BMI, median (IQR) kg/m^2^	27.3 (24.9–32.9)	27.5 (23.5–31.6)
Disease duration, median (IQR) years	14.0 (6.7–19.4)	7.7 (3.6–16.04)
RF positive^b^	16 (64.0)	72 (72.0)
Anti‐CCP positive^b^	21 (75.0)	94 (75.0)
ESR, median (IQR) mm/hour	22.5 (11.0–39)	27.5 (19.5–46.5)
CRP, median (IQR) mg/liter	9.0 (1.6–17.1)	11.2 (3.7–27.5)
DAS28, mean ± SD	5.7 ± 0.6	5.7 ± 0.9
Erosive disease^b^	11 (55)	63 (66.3)
DMARD therapy		
Prior biologic agent	2 (6.5)	12 (9.3)
Methotrexate	14 (45.1)	75 (58.1)
Median (IQR) mg/week	15 (10–20)	20 (15–25)
Sulfasalazine	8 (34.7)	22 (20.6)
Median (IQR) mg/day	1,000 (1,000–1,500)	1,000 (1,000–2,000)
Leflunomide	2 (6.6)	8 (6.2)
Hydroxychloroquine	0 (0)	5 (3.9)

a The disease‐modifying antirheumatic drugs (DMARDs) shown are those that were used most frequently by patients in this cohort. There were no significant differences between the groups except for disease duration (*P* < 0.03) and methotrexate dosage (*P* < 0.012). Except where indicated otherwise, values are the number (%). BMI = body mass index; IQR = interquartile range; RF = rheumatoid factor; anti‐CCP = anti–cyclic citrullinated peptide; ESR = erythrocyte sedimentation rate; CRP = C‐reactive protein; DAS28 = Disease Activity Score in 28 joints.

b Values are the number (%) of patients with nonmissing data.

In the GEE univariate model, the adalimumab drug level and antidrug antibody status were significantly associated with change in the DAS28 at 12 months (regression coefficient 0.078 [95% confidence interval (95% CI) 0.044, 0.11], *P* < 0.0001 and regression coefficient −0.76 [95% CI −1.24, −0.27], *P* = 0.002, respectively) (Table 3). The regression coefficient of −0.76 could be interpreted as the “pooled” difference in change in the DAS28 between patients with and those without antibodies over all followup time points, while a negative regression coefficient value suggests an inverse relationship between antidrug antibodies and improvement (i.e., change in the DAS28). Ordinal logistic regression further confirmed a positive association between the EULAR response at 12 months and the adalimumab level but a negative relationship between the EULAR response at 12 months and antidrug antibody status (Table 3).

**Table 3:** Association between treatment response and adalimumab levels/antidrug antibody status^a^

Variable	Regression coefficient (95% CI)	*P*
Univariate analysis using GEE (*Δ*DAS28)		
Adalimumab drug level	0.078 (0.044, 0.11)	<0.0001
Antidrug antibody status	−0.76 (−1.24, −0.27)	0.002
Age‐ and sex‐adjusted model using GEE (*Δ*DAS28)		
Adalimumab drug level	0.086 (0.05, 0.12)	<0.0001
Antidrug antibody status	−0.80 (−1.25, −0.34)	0.001
Multivariate model using GEE (*Δ*DAS28)
Adalimumab drug level	0.060 (0.015, 0.10)	0.009
Antidrug antibody status	−0.22 (−0.78, −0.33)	0.47
EULAR response at 12 months using ordinal logistic regression^b^		
Adalimumab drug level	0.11 (0.031, 0.20)	0.007
Antidrug antibody status (>12 AU/ml)	−1.03 (−1.99, −0.063)	0.037

a 95% CI = 95% confidence interval; GEE = generalized estimating equation; *Δ*DAS28 = change in the Disease Activity Score in 28 joints.

b All patients were categorized as having a good, moderate, or no response according to the European League Against Rheumatism (EULAR) criteria at 12 months.

In the multivariable GEE model, after adjusting for the confounders body mass index (BMI), disease duration, age, sex, and the time‐varying confounder adherence, the relationship between the adalimumab level and change in the DAS28 remained significant (regression coefficient 0.060 [95% CI 0.015, 0.10], *P* = 0.009). A subgroup analysis was performed in the 55.6% of patients in the methotrexate group, with adjustment for methotrexate dose and the above variables, and the significant association of adalimumab drug level with change in the DAS28 was maintained (regression coefficient 0.076 [95% CI 0.028, 0.12], *P* = 0.002).

The concentration–effect curve for treatment with adalimumab showed that a drug concentration of <5 μg/ml was associated with a lower change in the DAS28 (see Supplementary Figure 1, available on the *Arthritis & Rheumatology* web site at http://onlinelibrary.wiley.com/doi/10.1002/art.39169/abstract), similar to a prior study using adalimumab trough levels that identified a 5–8 μg/ml therapeutic window 25. ROC curve analysis was used to quantify the predictive value of testing random drug and antidrug antibody levels at 3 months on determining an outcome of no EULAR response at 12 months. The detection of low adalimumab levels (<5 μg/ml) at 3 months was associated with an AUC of 0.66 (95% CI 0.55, 0.77), and detection of antidrug antibodies at 3 months was associated with an AUC of 0.68 (95% CI 0.54, 0.81). The presence of both low adalimumab levels and antidrug antibodies at 3 months was associated with an AUC of 0.71 (95% CI 0.57, 0.85).

In 20 samples (from 13 patients), the random mean adalimumab drug level was <0.1 μg/ml (0 μg/ml in 13 samples). This was significantly associated with no EULAR response, using logistic regression (regression coefficient 2.29 [95% CI 1.13, 3.44], *P* < 0.0001) at the corresponding time point. Interestingly, a good EULAR response was observed at >1 sequential time point in 2 patients with an adalimumab level of <0.1 μg/ml. At 6 months and 12 months, the first patient had antidrug antibody levels of 430 AU/ml and 400 AU/ml, respectively, and the second patient had antidrug antibody levels of 1,500 AU/ml and 9,100 AU/ml, respectively. Both patients were receiving adalimumab monotherapy (and were not receiving methotrexate), suggesting that these patients may have attained spontaneous remission and therefore no longer required the presence of drug to achieve a good response.

### Antidrug antibodies against etanercept, etanercept levels, and association with treatment response

After 3, 6, and 12 months of etanercept treatment, etanercept antibodies were not detected in any of the tested patients. Etanercept levels were measured in 421 samples from 171 patients over 12 months. The levels of etanercept were associated with a EULAR response at 12 months in the univariate ordinal logistic regression model (regression coefficient 0.088 [95% CI 0.019, 0.16], *P* = 0.012), and this association remained significant after adjustment for age and sex (Table 4). However, the association between treatment response and etanercept levels lost significance in the univariate GEE model and also in the logistic regression model after adjusting for confounders including age, sex, BMI, disease duration, and adherence (Table 4). Although a trend toward higher drug levels at 12 months was shown to be associated with a good EULAR response at 12 months (see Supplementary Figure 2, available on the *Arthritis & Rheumatology* web site at http://onlinelibrary.wiley.com/doi/10.1002/art.39169/abstract), the concentration***–***effect curve for etanercept did not identify a clear therapeutic window to indicate the level of the optimal treatment response (from concentration–effect curves) (see Supplementary Figure 1, available on the *Arthritis & Rheumatology* web site at http://onlinelibrary.wiley.com/doi/10.1002/art.39169/abstract). Furthermore, ROC curve analysis to quantify the value of low etanercept non‐trough levels (<5 μg/ml) at 3 months for predicting no EULAR response at 12 months revealed an AUC of 0.51 (95% CI 0.41, 0.61). A lower etanercept level cutoff of <3.23 μg/ml (see Supplementary Figure 1, available on the *Arthritis & Rheumatology* web site at http://onlinelibrary.wiley.com/doi/10.1002/art.39169/abstract) at 3 months resulted in an AUC of 0.58 (95% CI 0.46, 0.70), suggesting poor value of etanercept non‐trough levels for predicting no EULAR response at 12 months.

**Table 4:** Association of the treatment response with etanercept levels^a^

	Regression coefficient (95% CI)	*P*
Univariate analysis using GEE
Etanercept level	0.0080 (−0.51, 0.031)	0.51
EULAR response at 12 months using ordinal logistic regression (univariate analysis)
Etanercept level	0.088 (0.019, 0.16)	0.012
EULAR response at 12 months, age‐ and sex‐adjusted, using ordinal logistic regression
Etanercept level	0.081 (0.011, 0.15)	0.022
EULAR response at 12 months multivariate‐adjusted model, using ordinal logistic regression^b^
Etanercept level	0.057 (−0.050, 0.16)	0.30

a All patients were categorized as having a good, moderate, or no response according to the European League Against Rheumatism (EULAR) criteria. 95% CI = 95% confidence interval; GEE = generalized estimating equation.

b Adjusted for age, sex, body mass index, disease duration, and adherence.

### Predictors of low drug levels in patients treated with adalimumab or etanercept

We used a GEE model to test the longitudinal association of adalimumab levels and antidrug antibodies over 12 months (with adalimumab levels as the dependent variable and antidrug antibody status as the predictor). This demonstrated a strong inverse association between the 2 factors (regression coefficient −4.77 [95% CI −6.39, −3.15], *P* < 0.0001). To identify additional predictors of low drug levels in all patients (the adalimumab and etanercept groups combined) over 12 months and to obtain an adjusted estimate of the effect of antidrug antibody status on drug level, a logistic regression model for repeated measures (GEE with a logit link for binary outcomes) was used. Thresholds for low adalimumab and etanercept levels were determined using the generated concentration***–***effect curves (see Supplementary Figure 1, available on the *Arthritis & Rheumatology* web site at http://onlinelibrary.wiley.com/doi/10.1002/art.39169/abstract).

After adjustment for multiple confounders, the strongest predictor of low drug levels over time (in patients receiving adalimumab) continued to be antidrug antibody status (regression coefficient 1.27 [95% CI 0.44, 2.07], *P* = 0.003). Self‐reported adherence data were available for 80.4% of adalimumab‐treated patients and 73.1% of etanercept‐treated patients at all time points over 12 months. Adherence and BMI were also shown to be significant predictors in the fully adjusted model (Table 5). In the combined GEE model including adalimumab and etanercept, a BMI of ≥30 kg/m^2^ was associated with low drug levels (regression coefficient 0.78 [95% CI 0.37, 1.18], *P* < 0.0001).

**Table 5:** Predictors of low drug levels in adalimumab‐ and etanercept‐treated patients, using GEE for binary outcomes^a^

Variable	Coefficient (95% CI)	*P*
Age	0.0056 (−0.20, −0.031)	0.67
Sex	0.054 (−0.58, −0.69)	0.87
Body mass index	0.055 (0.017, 0.094)	0.005
Baseline disease activity^b^	0.15 (−0.21, −0.50)	0.43
Methotrexate use	−0.15 (−0.67, 0.38)	0.59
Antidrug antibody status^c^	1.27 (0.44, 2.07)	0.003
Adherence	−0.68 (−1.29, −0.07)	0.028

a A low adalimumab level was defined as <5 μg/ml, and a low etanercept level was defined as <3.62 μg/ml. GEE = generalized estimating equation; 95% CI = 95% confidence interval.

b Based on the Disease Activity Score in 28 joints.

c Adalimumab‐treated patients only.

## DISCUSSION

Our study is the first to demonstrate that adalimumab drug levels and antidrug antibody status, ascertained from non–trough‐level serum samples, are useful for the early prediction of a EULAR response at 12 months. Of the 2 variables, the adalimumab drug level was the better predictor of treatment response, after adjustment for confounding variables. Etanercept drug levels, tested in non–trough‐level serum samples, had a wider variation over 12 months of treatment and were less useful as a predictor of the future treatment response. BMI and adherence appeared to be significant predictors of drug levels in both adalimumab‐ and etanercept‐treated patients.

The main strengths of the study include a large sample size, prospective serial sampling, a well‐characterized cohort of patients, and availability of other outcome measures such as self‐reported adherence. Because serum samples were obtained during routine clinic visits, as opposed to trough serum samples, a pragmatic approach to immunogenicity/drug level testing can be evaluated because, in a clinical setting, it may not always be possible to obtain serum just before the patient is due to receive the next injection. We chose to test for immunogenicity using RIA, because this method is less prone to drug interference than the more commonly used bridging ELISA method. However, in the presence of circulating drug levels, RIA may still be prone to drug interference, and it should be noted that in our study antidrug antibodies to adalimumab were detected in a lower number of RA patients compared with previous estimates using the same assay 5. In the future, newer techniques, such as pH‐shift–anti‐idiotype antigen‐binding tests (pH dissociation assays that are based on acid dissociation of adalimumab–antidrug antibody complexes and therefore are more sensitive in the presence of free drug), may be more useful in this setting 20.

The results of the current study are consistent with those of several previous studies that confirmed that treatment failure is higher in patients in whom antidrug antibodies to monoclonal antibodies such as adalimumab and infliximab develop 6, 26. The underlying mechanism is thought to be either increased drug clearance or neutralization of the active component of the protein 27, 28. It appears that the effect of antidrug antibodies on the drug level is of the most clinical significance. We showed an inverse correlation between antidrug antibodies and drug levels, suggesting that measurement of antidrug antibodies may be useful to determine the etiology of low adalimumab non‐trough levels, therefore facilitating the decision regarding the next therapeutic option in nonresponding patients, similar to previously published algorithms 29, 30. In keeping with a previous study, median methotrexate doses were lower in patients in whom immunogenicity developed compared with patients in whom immunogenicity did not develop 31. This suggests that both continued treatment with methotrexate and anti‐TNF therapy 11 as well as administration of methotrexate at the maximum tolerated dose may be important in preventing immunogenicity and future loss of response.

Previous studies using RIAs and ELISAs also failed to detect antidrug antibodies to etanercept 23, 24, 32, and in those studies in which these antibodies were observed, antidrug antibodies had no apparent effect on treatment response or clinical outcome 33, 34, 35. Low trough concentrations of etanercept have previously been associated with a poor response to treatment in patients with RA treated with etanercept for up to 6 months 23. In our study, etanercept levels were associated with a EULAR response at 12 months (by univariate analysis only); however, there was not a clear cutoff to identify a therapeutic concentration range. A similar wide variation in the concentration–effect curve was also seen recently in an ankylosing spondylitis cohort study that measured etanercept trough levels 36. This variability may be attributable to the short half‐life of etanercept (100 hours), suggesting that pre‐dose sampling is particularly important when interpreting the results for this drug.

Several extrinsic and intrinsic factors may also influence the pharmacokinetics of anti‐TNF agents, such as BMI (higher weight is associated with a larger central volume of distribution), sex (which affects volume/distribution, with shorter half‐lives in female patients) 37, adherence, disease activity, and albumin levels (high disease activity and low albumin levels are associated with accelerated drug clearance). A major strength of the current study design was that each of these factors (except albumin) was recorded for our patients, but the association with drug levels remained significant even after adjustment for these confounders in adalimumab‐treated patients.

In a clinical setting, testing for adalimumab levels could inform practice. In the absence of antidrug antibodies, a low drug level may be attributable to a high BMI and therefore volume of distribution. BMI has been described as a factor affecting anti‐TNF levels 23, 36; however, previously reported data have been inconclusive. A BMI cutoff of ≥30 kg/m^2^ in our patients was determined as being significant enough to be associated with lower drug levels in both the adalimumab and etanercept cohorts. Patients with high BMIs may fail to respond not only due to issues of bioavailability/volume of distribution leading to a lower drug level, but also due to the inflammatory effect of adipose tissue influencing the target load 38. Using an anti‐TNF agent that accounts for patient weight (e.g., switching to an intravenous alternative) or escalating the dose of subcutaneous therapy may be a more effective strategy in patients with a high BMI; however, this notion needs to be fully explored in a randomized controlled trial.

In patients with a low drug level, a normal BMI, and no antidrug antibodies, the clinician may prompt a discussion with the patient regarding adherence to the medication; we observed that poor adherence or nonadherence was a predictor of low non‐trough levels and also has previously been associated with a poor treatment response 18. Furthermore, 2 patients in our study achieved a good EULAR response at 6 months and 12 months, in the presence of undetectable adalimumab levels in association with high‐titer antidrug antibodies. This would suggest that their disease had entered spontaneous remission, obviating the need for continuation of treatment with an anti‐TNF agent. Therefore, this situation represents a unique opportunity for stopping treatment with these expensive drugs in such responding patients.

In conclusion, in the context of drug non‐trough levels, measurement of adalimumab levels and antidrug antibodies early in the treatment course is predictive of subsequent treatment response. Consideration of these factors, along with BMI and adherence, may help when deciding the best treatment strategy in patients in whom adalimumab does not appear to be effective. To maximize the chances of efficacy, methotrexate treatment should be maintained at the highest dose the patient can tolerate. Etanercept non‐trough levels, while associated with treatment response, were less predictive and unlikely to be clinically useful in isolation.

## AUTHOR CONTRIBUTIONS

All authors were involved in drafting the article or revising it critically for important intellectual content, and all authors approved the final version to be published. Dr. Barton had full access to all of the data in the study and takes responsibility for the integrity of the data and the accuracy of the data analysis.

### Study conception and design

Jani, Chinoy, Warren, Griffiths, Morgan, Wilson, Isaacs, Hyrich, Barton.

### Acquisition of data

Jani, Plant, Barton.

### Analysis and interpretation of data

Jani, Chinoy, Warren, Griffiths, Plant, Fu, Morgan, Isaacs, Hyrich, Barton.

## Supporting information

Supporting Information

## THE BRAGGSS COLLABORATORS

The BRAGGSS Collaborators, in addition to authors Chinoy, Morgan, Isaacs, Hyrich, and Barton, are as follows: P. J. Prouse, R. K. Moitra, D. J. Shawe, M. Nisar, K. Fairburn, J. Nixon, T. Barnes, M. Hui, D. Coady, D. Wright, C. Morley, G. Raftery, C. Bracewell, M. Bridges, D. Armstrong, A. J. Chuck, S. Hailwood, N. Kumar, D. Ashok, R. Reece, S. C. O'Reilly, T. Ding, L. J. Badcock, C. M. Deighton, N. Raj, M. R. Regan, G. D. Summers, R. A. Williams, J. R. Lambert, R. Stevens, C. Wilkinson, C. A. Kelly, J. Hamilton, C. R. Heycock, V. Saravanan, A. Cope, T. Garrood, N. Ng, B. Kirkham, M. Green, A. Gough, C. Lawson, D. Das, E. Borbas, T. Wazir, P. Emery, S. Bingham, H. A. Bird, P. G. Conaghan, C. T. Pease, R. J. Wakefield, M. Buch, I. Bruce, R. Gorodkin, P. Ho, B. Parker, W. Smith, E. Jenkins, C. Mukhtyar, K. Gaffney, A. J. Macgregor, T. Marshall, P. Merry, C. DeSilva, F. N. Birrell, P. R. Crook, B. Szebenyi, D. Bates, D. James, T. Gillott, A. Alvi, C. Grey, J. Browning, J. F. McHale, I. C. Gaywood, A. C. Jones, P. Lanyon, I. Pande, M. Doherty, A. Gupta, P. A. Courtney, A. Srikanth, A. Abhishek, L. Das, M. Pattrick, H. N. Snowden, A. P. Bowden, E. E. Smith, P. Klimiuk, D. J. Speden, S. Naz, J. M. Ledingham, R. G. Hull, F. McCrae, A. Cooper, S. A. Young‐Min, E. Wong, R. Shaban, A. D. Woolf, M. Davis, D. Hutchinson, A. Endean, D. Mewar, E. J. Tunn, K. Nelson, T. D. Kennedy, C. Dubois, J. Pauling, E. Korendowych, T. Jenkinson, R. Sengupta, A. Bhalla, N. McHugh, T. O'Neil, A. L. Herrick, A. K. Jones, R. G. Cooper, W. G. Dixon, B. Harrison, C. D. Buckley, D. C. Carruthers, R. Elamanchi, P. C. Gordon, K. A. Grindulis, F. Khattak, K. Raza, K. Situnayake, M. Akil, S. Till, L. Dunkley, R. Tattersall, R. Kilding, T. Tait, J. Maxwell, S. Till, K.‐P. Kuet, M. J. Plant, F. Clarke, J. N. Fordham, S. Tuck, S. K. Pathare, A. Paul, C. P. Marguerie, S. P. Rigby, N. Dunn, I. Abbas, C. Filer, V. E. Abernethy, A. R. Clewes, J. K. Dawson, G. Kitas, N. Erb, R. Klocke, A. J. Whallett, K. Douglas, A. Pace, R. Sandhu, H. John, L. Shand, S. Lane, H. Foster, B. Griffiths, I. Griffiths, L. Kay, W.‐F. Ng, P. N. Platt, D. J. Walker, P. Peterson, A. Lorenzi, M. Friswell, B. Thompson, M. Lee, A. Pratt, N. D. Hopkinson, C. A. Dunne, B. Quilty, J. Marks, S. Mukherjee, D. Mulherin, S. V. Chalam, T. Price, T. Sheeran, S. Venkatachalam, S. Baskar, W. Al‐Allaf, F. McKenna, P. Shah, A. Filer, S. J. Bowman, P. Jobanputra, E. C. Rankin, M. Allen, K. Chaudhuri, S. Dubey, A. Price‐Forbes, J. Ravindran, A. Samanta, P. Sheldon, W. Hassan, J. Francis, A. Kinder, R. Neame, A. Moorthy, M. Bukhari, L. Ottewell, E. Palkonyai, S. Hider, A. Hassell, A. Menon, C. Dowson, S. Kamath, J. Packham, S. Dutta, S. Price, E. Roddy, Z. Paskins, D. T. O'Reilly, V. Rajagopal, S. Bhagat, C. B. Chattopadhyay, M. Green, D. Quinn, A. Isdale, A. Brown, B. Saleem, B. Foo, Z. Al Saffar, G. Koduri.

## References

[1] Weinblatt ME , Kremer JM , Bankhurst AD , Bulpitt KJ , Fleischmann RM , Fox RI , et al. A trial of etanercept, a recombinant tumor necrosis factor receptor:Fc fusion protein, in patients with rheumatoid arthritis receiving methotrexate. N Engl J Med 1999;340:253–9. 9920948 10.1056/NEJM199901283400401

[2] Weinblatt ME , Keystone EC , Furst DE , Moreland LW , Weisman MH , Birbara CA , et al. Adalimumab, a fully human anti–tumor necrosis factor α monoclonal antibody, for the treatment of rheumatoid arthritis in patients taking concomitant methotrexate: the ARMADA trial. Arthritis Rheum 2003;48:35–45. 12528101 10.1002/art.10697

[3] Finckh A , Simard JF , Gabay C , Guerne PA. Evidence for differential acquired drug resistance to anti‐tumour necrosis factor agents in rheumatoid arthritis. Ann Rheum Dis 2006;65:746–52. 16339288 10.1136/ard.2005.045062 PMC1798167

[4] Radstake TR , Svenson M , Eijsbouts AM , van den Hoogen FH , Enevold C , van Riel PL , et al. Formation of antibodies against infliximab and adalimumab strongly correlates with functional drug levels and clinical responses in rheumatoid arthritis. Ann Rheum Dis 2009;68:1739–45. 19019895 10.1136/ard.2008.092833

[5] Bartelds GM , Krieckaert CL , Nurmohamed MT , van Schouwenburg PA , Lems WF , Twisk JW , et al. Development of antidrug antibodies against adalimumab and association with disease activity and treatment failure during long‐term follow‐up. JAMA 2011;305:1460–8. 21486979 10.1001/jama.2011.406

[6] Garces S , Demengeot J , Benito‐Garcia E. The immunogenicity of anti‐TNF therapy in immune‐mediated inflammatory diseases: a systematic review of the literature with a meta‐analysis. Ann Rheum Dis 2013;72:1947–55. 23223420 10.1136/annrheumdis-2012-202220

[7] Maneiro JR , Salgado E , Gomez‐Reino JJ. Immunogenicity of monoclonal antibodies against tumor necrosis factor used in chronic immune‐mediated inflammatory conditions: systematic review and meta‐analysis. JAMA Intern Med 2013;173:1416–28. 23797343 10.1001/jamainternmed.2013.7430

[8] Paramarta JE , Baeten DL. Adalimumab serum levels and antidrug antibodies towards adalimumab in peripheral spondyloarthritis: no association with clinical response to treatment or with disease relapse upon treatment discontinuation. Arthritis Res Ther 2014;16:R160. 25074046 10.1186/ar4675 PMC4261980

[9] Smolen JS , Landewe R , Breedveld FC , Buch M , Burmester G , Dougados M , et al. EULAR recommendations for the management of rheumatoid arthritis with synthetic and biological disease‐modifying antirheumatic drugs: 2013 update. Ann Rheum Dis 2014;73:492–509. 24161836 10.1136/annrheumdis-2013-204573 PMC3933074

[10] Schellekens H. Bioequivalence and immunogenicity of biopharmaceuticals. Nat Rev Drug Discov 2002;1:457–62. 12119747 10.1038/nrd818

[11] Jani M , Barton A , Warren RB , Griffiths CE , Chinoy H. The role of DMARDs in reducing the immunogenicity of TNF inhibitors in chronic inflammatory diseases. Rheumatology (Oxford) 2014;53:213–22. 23946436 10.1093/rheumatology/ket260 PMC3894670

[12] Hart MH , de Vrieze H , Wouters D , Wolbink GJ , Killestein J , de Groot ER , et al. Differential effect of drug interference in immunogenicity assays. J Immunol Methods 2011;372:196–203. 21824477 10.1016/j.jim.2011.07.019

[13] Krieckaert CL , Jamnitski A , Nurmohamed MT , Kostense PJ , Boers M , Wolbink G. Comparison of long‐term clinical outcome with etanercept treatment and adalimumab treatment of rheumatoid arthritis with respect to immunogenicity. Arthritis Rheum 2012;64:3850–5. 22933315 10.1002/art.34680

[14] Bendtzen K , Geborek P , Svenson M , Larsson L , Kapetanovic MC , Saxne T. Individualized monitoring of drug bioavailability and immunogenicity in rheumatoid arthritis patients treated with the tumor necrosis factor α inhibitor infliximab. Arthritis Rheum 2006;54:3782–9. 17133559 10.1002/art.22214

[15] Potter C , Cordell HJ , Barton A , Daly AK , Hyrich KL , Mann DA , et al. Association between anti‐tumour necrosis factor treatment response and genetic variants within the TLR and NFκB signalling pathways. Ann Rheum Dis 2010;69:1315–20. 20448286 10.1136/ard.2009.117309

[16] Arnett FC , Edworthy SM , Bloch DA , McShane DJ , Fries JF , Cooper NS , et al. The American Rheumatism Association 1987 revised criteria for the classification of rheumatoid arthritis. Arthritis Rheum 1988;31:315–24. 3358796 10.1002/art.1780310302

[17] Prevoo ML , van't Hof MA , Kuper HH , van Leeuwen MA , van de Putte LB , van Riel PL . Modified disease activity scores that include twenty‐eight–joint counts: development and validation in a prospective longitudinal study of patients with rheumatoid arthritis. Arthritis Rheum 1995;38:44–8. 7818570 10.1002/art.1780380107

[18] Bluett J , Morgan C , Thurston L , Plant D , Hyrich KL , Morgan AW , et al. Impact of inadequate adherence on response to subcutaneously administered anti‐tumour necrosis factor drugs: results from the Biologics in Rheumatoid Arthritis Genetics and Genomics Study Syndicate cohort. Rheumatology (Oxford) 2015;54:494–9. 25213131 10.1093/rheumatology/keu358 PMC4334684

[19] Van Gestel AM , Prevoo ML , van 't Hof MA , van Rijswijk MH , van de Putte LB , van Riel PL. Development and validation of the European League Against Rheumatism response criteria for rheumatoid arthritis. Comparison with the preliminary American College of Rheumatology and the World Health Organization/International League Against Rheumatism Criteria. Arthritis Rheum 1996;39:34–40. 8546736 10.1002/art.1780390105

[20] Van Schouwenburg PA , Bartelds GM , Hart MH , Aarden L , Wolbink GJ , Wouters D. A novel method for the detection of antibodies to adalimumab in the presence of drug reveals “hidden” immunogenicity in rheumatoid arthritis patients. J Immunol Methods 2010;362:82–8. 20833178 10.1016/j.jim.2010.09.005

[21] Sethu S , Govindappa K , Alhaidari M , Pirmohamed M , Park K , Sathish J. Immunogenicity to biologics: mechanisms, prediction and reduction. Arch Immunol Ther Exp (Warsz) 2012;60:331–44. 22930363 10.1007/s00005-012-0189-7

[22] Aalberse RC , Stapel SO , Schuurman J , Rispens T. Immunoglobulin G4: an odd antibody. Clin Exp Allergy 2009;39:469–77. 19222496 10.1111/j.1365-2222.2009.03207.x

[23] Jamnitski A , Krieckaert CL , Nurmohamed MT , Hart MH , Dijkmans BA , Aarden L , et al. Patients non‐responding to etanercept obtain lower etanercept concentrations compared with responding patients. Ann Rheum Dis 2012;71:88–91. 21914626 10.1136/annrheumdis-2011-200184

[24] Hoshino M , Yoshio T , Onishi S , Minota S. Influence of antibodies against infliximab and etanercept on the treatment effectiveness of these agents in Japanese patients with rheumatoid arthritis. Mod Rheumatol 2012;22:532–40. 22173229 10.1007/s10165-011-0567-8

[25] Pouw MF , Krieckaert CL , Nurmohamed MT , van der Kleij D , Aarden L , Rispens T , et al. Key findings towards optimising adalimumab treatment: the concentration‐effect curve. Ann Rheum Dis 2015;74:513–8. 24326008 10.1136/annrheumdis-2013-204172

[26] Vincent FB , Morand EF , Murphy K , Mackay F , Mariette X , Marcelli C. Antidrug antibodies (ADAb) to tumour necrosis factor (TNF)‐specific neutralising agents in chronic inflammatory diseases: a real issue, a clinical perspective. Ann Rheum Dis 2013;72:165–78. 23178294 10.1136/annrheumdis-2012-202545

[27] Van der Laken CJ , Voskuyl AE , Roos JC , Stigter van Walsum M , de Groot ER , Wolbink G , et al. Imaging and serum analysis of immune complex formation of radiolabelled infliximab and anti‐infliximab in responders and non‐responders to therapy for rheumatoid arthritis. Ann Rheum Dis 2007;66:253–6. 16793840 10.1136/ard.2006.057406 PMC1798492

[28] Van Schouwenburg PA , van de Stadt LA , de Jong RN , van Buren EE , Kruithof S , de Groot E , et al. Adalimumab elicits a restricted anti‐idiotypic antibody response in autoimmune patients resulting in functional neutralisation. Ann Rheum Dis 2013;72:104–9. 22759910 10.1136/annrheumdis-2012-201445

[29] Bendtzen K. Is there a need for immunopharmacologic guidance of anti‐tumor necrosis factor therapies? Arthritis Rheum 2011;63:867–70. 21452309 10.1002/art.30207

[30] Jamnitski A , Bartelds GM , Nurmohamed MT , van Schouwenburg PA , van Schaardenburg D , Stapel SO , et al. The presence or absence of antibodies to infliximab or adalimumab determines the outcome of switching to etanercept. Ann Rheum Dis 2011;70:284–8. 21068090 10.1136/ard.2010.135111

[31] Krieckaert CL , Nurmohamed MT , Wolbink GJ. Methotrexate reduces immunogenicity in adalimumab treated rheumatoid arthritis. Ann Rheum Dis 2012;71:1914–5. 22586169 10.1136/annrheumdis-2012-201544

[32] De Vries MK , van der Horst‐Bruinsma IE , Nurmohamed MT , Aarden LA , Stapel SO , Peters MJ , et al. Immunogenicity does not influence treatment with etanercept in patients with ankylosing spondylitis. Ann Rheum Dis 2009;68:531–5. 18375542 10.1136/ard.2008.089979

[33] Klareskog L , Gaubitz M , Rodriguez‐Valverde V , Malaise M , Dougados M , Wajdula J. Assessment of long‐term safety and efficacy of etanercept in a 5‐year extension study in patients with rheumatoid arthritis. Clin Exp Rheumatol 2011;29:238–47. 21418785

[34] Keystone EC , Schiff MH , Kremer JM , Kafka S , Lovy M , DeVries T , et al. Once‐weekly administration of 50 mg etanercept in patients with active rheumatoid arthritis: results of a multicenter, randomized, double‐blind, placebo‐controlled trial. Arthritis Rheum 2004;50:353–63. 14872476 10.1002/art.20019

[35] Dore RK , Mathews S , Schechtman J , Surbeck W , Mandel D , Patel A , et al. The immunogenicity, safety, and efficacy of etanercept liquid administered once weekly in patients with rheumatoid arthritis. Clin Exp Rheumatol 2007;25:40–6. 17417989

[36] Kneepkens EL , Krieckaert CL , van der Kleij D , Nurmohamed MT , van der Horst‐Bruinsma IE , Rispens T , et al. Lower etanercept levels are associated with high disease activity in ankylosing spondylitis patients at 24 weeks of follow‐up. Ann Rheum Dis 2014. E‐pub ahead of print. 10.1136/annrheumdis-2014-205213 24812290

[37] Mahil SK , Arkir Z , Richards G , Lewis CM , Barker JN , Smith CH. Predicting treatment response in psoriasis using serum levels of adalimumab and etanercept: a single centre, cohort study. Br J Dermatol 2013;169:306–13. 23550925 10.1111/bjd.12341

[38] Tilg H , Moschen AR. Adipocytokines: mediators linking adipose tissue, inflammation and immunity. Nat Rev Immunol 2006;6:772–83. 16998510 10.1038/nri1937

