# Supplementary material for: Clinical Utility of Random Anti–Tumor Necrosis Factor Drug–Level Testing and Measurement of Antidrug Antibodies on the Long‐Term Treatment Response in Rheumatoid Arthritis
Source: Arthritis Rheumatol. 2015 Jul 28;67(8):2011–9. doi: 10.1002/art.39169 (PMC4843946; doi:10.1002/art.39169)
Supplement: Supplementary file 1 — Supporting Information [file ART-67-2011-s001.doc]

# SUPPLEMENTARY MATERIAL

**Supplementary Table 1: Adalimumab drug levels in patients stratified by anti-drug antibody status**

| **Time point** | **3 months** | | **6 months** | | **12 months** | |
| --- | --- | --- | --- | --- | --- | --- |
|  | Mean (SD) | Median (IQR) | Mean (SD) | Median (IQR) | Mean (SD) | Median (IQR) |
| **Adalimumab drug level if ADAb -ve (µg/mL)** | 10.8 (2.6) | 12.0 (11.4-12.0) | 11.2 (2.1) | 12.0 (11.9-12) | 9.6 (3.8) | 12.0 (7.9-12) |
| **Adalimumab drug level if ADAb +ve (µg/mL)** | 4.9 (4.2) | 4.6 (0.9-8.2) | 4.5 (4.9) | 2.1(0-8.7) | 3.9 (4.4) | 1.7 (0-6.8) |
| **ADAb level AU/ml*** | 80.1 (91.9) | 37.0 (23-95) | 703.2 (1,943) | 48.5 (18-200) | 8,632 .0 (26,873) | 25.0 (21-2,800) |

Abbreviations: ADAb, anti-drug antibodies; AU arbitrary units. Adalimumab drug levels could be detected up to a maximal concentration of 12 µg/mL. *ADAb level in patients who were positive for anti-drug antibodies.

**Supplementary Figure 1: Concentration effect curve for adalimumab and etanercept patients**

**
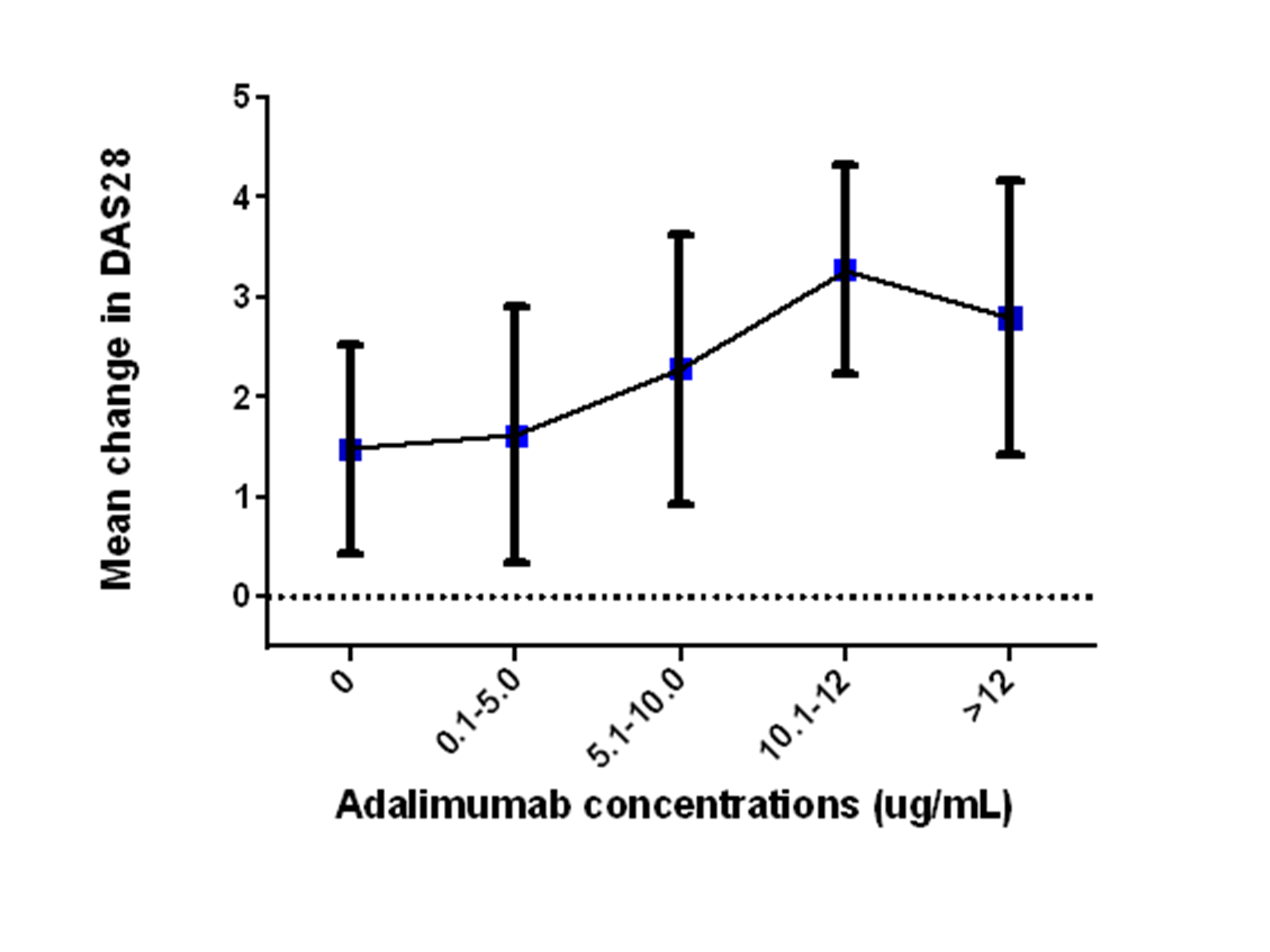
**


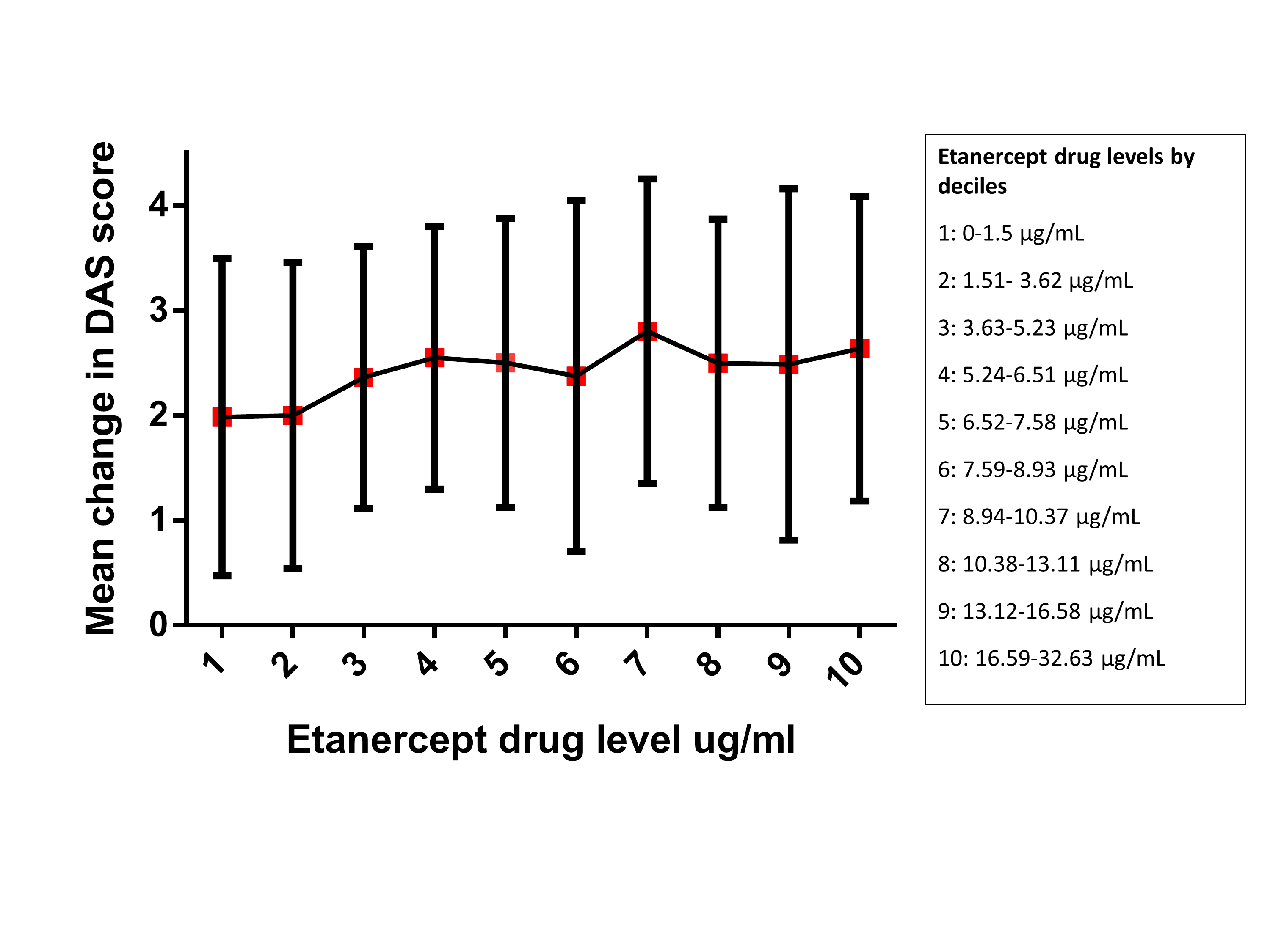


Adalimumab and etanercept concentration-effect curves in rheumatoid arthritis patients treated over 12 months. Abbreviations: DAS score, disease activity of 28 joints. One dot represents the mean ΔDAS28 per samples in the quantile or decile.

**Supplementary Figure 2: Association of etanercept drug levels with good EULAR response at 12 months using logistic regression – adjusted predictions with 95% confidence intervals**

**
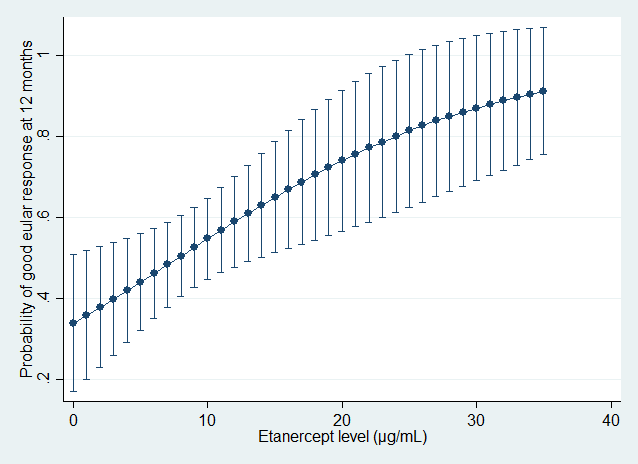
**
